# Storage of packed red blood cells impairs an inherent coagulation property of erythrocytes

**DOI:** 10.3389/fphys.2022.1021553

**Published:** 2022-11-25

**Authors:** Thomas Öhlinger, Ernst W. Müllner, Magdalena Fritz, Maike Werning, Joanna Baron-Stefaniak, Christof Jungbauer, David M. Baron, Ulrich Salzer

**Affiliations:** ^1^ Center for Medical Biochemistry, Max Perutz Labs (MPL), Medical University of Vienna, Vienna, Austria; ^2^ Department of Anaesthesia, Intensive Care Medicine and Pain Medicine, Medical University of Vienna, Vienna, Austria; ^3^ Blood Service for Vienna, Lower Austria and Burgenland, Austrian Red Cross, Vienna, Austria

**Keywords:** packed red blood cells, clotting, transfusion, microvesicles, thromboelastometry, erythrocytes, clotting time, clot formation time

## Abstract

Storage of packed red blood cells is associated with changes in erythrocytes that over time increasingly impair cellular function and potentially contribute to adverse effects associated with blood transfusion. Exposure of phosphatidylserine at the outer membrane leaflet of erythrocytes and shedding of microvesicles (MVs) during packed red blood cell storage are alterations assumed to increase the risk of prothrombotic events in recipients. Here, we used rotational thromboelastometry to study the coagulation process in blood samples with erythrocytes from stored PRBCs reconstituted with freshly prepared platelet-rich plasma. We explored the influence of following effects on the coagulation process: 1) PRBC storage duration, 2) differences between erythrocytes from stored PRBCs compared to freshly drawn erythrocytes, and 3) the contribution of added MVs. Interestingly, despite of a higher fraction of PS-positive cells, erythrocytes from PRBCs stored for 6 weeks revealed longer clotting times than samples with erythrocytes stored for 2 or 4 weeks. Further, clotting times and clot formation times were considerably increased in samples reconstituted with erythrocytes from stored PRBCs as compared to fresh erythrocytes. Moreover, MVs added to reconstituted samples elicited only comparably small and ambiguous effects on coagulation. Thus, this study provides no evidence for an amplified clotting process from prolonged storage of PRBCs but on the contrary implicates a loss of function, which may be of clinical significance in massive transfusion. Our observations add to the increasing body of evidence viewing erythrocytes as active players in the clotting process.

## Introduction

Over the course of routine 6-week cold storage in packed red blood cell (PRBC) units, erythrocytes progressively accumulate biochemical alterations, collectively termed “storage lesions”. These changes include irreversible lipid and protein modifications *via* glycation, altered energy metabolism, methemoglobin formation and increased oxidative stress. Some notable consequences are an impaired connectivity of the cytoskeleton, altered cation influx/efflux, reduced cell membrane deformability, changes in cell morphology and ultimately hemolysis (Yoshida et al., 2019). Adverse clinical effects have been associated with prolonged storage time of the PRBC units prior to transfusion. PRBC transfusion is for example associated with an increased risk of organ failure (Zallen et al., 1999), infection (Spadaro et al., 2017), hemolytic reactions (Vamvakas and Blajchman, 2010), alloimmunization (Desai et al., 2015) and thromboembolism (Dubovoy and Engoren, 2016). Such transfusion-related thromboembolic events have been suspected to be triggered by microparticles/microvesicles (MVs) that progressively accumulate in the PRCBs (Rubin et al., 2010). These storage-induced MVs are generated by an exovesiculation process of the plasma membrane, display distinct membrane protein composition, and contain high amounts of phosphatidylserine (PS) on their surface (Salzer et al., 2008). By exposing this negatively charged phospholipid, which is normally confined to the inner leaflet of the bilayer, and by their high membrane curvature, MVs provide an ideal surface for the assembly of factors of the clotting cascade. Phosphatidylserine is not only present at MVs, but also becomes increasingly exposed at the surface of PRBCs during prolonged storage (Lu et al., 2011; Dinkla et al., 2014; Mittag et al., 2015). In fact, both MVs (Salzer et al., 2008; Van Der Meijden et al., 2012; Rubin et al., 2013; Aung et al., 2017) and PRBCs (Lu et al., 2011; Dinkla et al., 2014; Mittag et al., 2015) exhibit prothrombotic characteristics as assessed by various assays *in vitro*. This implicates that both components of the storage unit might actually contribute to the increased *in vivo* risk of thromboembolism following transfusion.

For a long time, the role of erythrocytes in hemostasis has been regarded as rather passive and mainly ascribed to their high abundance in blood: 1) the rheological properties of blood flowing through the circulatory system ascertain that the bulk of erythrocytes pushes the smaller platelets towards the vessel walls and thereby assures quick sealing in case of vascular damage; 2) thrombus formation was assumed to be based on platelet activation and fibrin filament assembly to result in a locally growing meshwork that necessarily but rather accidently entraps erythrocytes passing by. However, several findings indicate that this view is too simplistic. There is evidence for direct interaction between platelets and erythrocytes *via* αIIbβ3–ICAM4 and reduced fibrin and thrombus formation upon blockage of this interaction (Hermand et al., 2003; Du et al., 2014). Erythrocytes have further been shown to modulate platelet function in several other ways (Rocca and FitzGerald, 1997; Weisel and Litvinov, 2019). Conversely, activated platelets can rapidly induce procoagulant properties in erythrocytes. Lysophosphatidic acid (LPA), a lipid mediator secreted by activated platelets, induces a signaling cascade in erythrocytes, which results in calcium influx (Yang et al., 2000), MV release (Chung et al., 2007) and PS exposure by activating the phospholipid scramblase TMEM16F (Ohlinger et al., 2020). These LPA-induced effects would clearly assign a coagulation-promoting role to erythrocytes likely during both thrombin generation and clot formation. Moreover, during clot contraction, the shape of entrapped erythrocytes is transformed into a polyhedron-like structure due to contractile forces exerted by platelets and the fibrin meshwork, thereby forming an impermeable barrier essential for wound healing (Cines et al., 2014). Thus, it is now assumed that erythrocytes actively contribute to coagulation, probably being involved at several steps of this complex process. However, while several pro- and anti-hemostatic effects of erythrocytes and their interactions with other cellular and plasma components of the coagulation machinery were assessed by assays *in vitro* (Weisel and Litvinov, 2019), it still remained largely unknown whether and if - to which extent - each of these effects is relevant for thrombus formation *in vivo*.

Rapid and accurate assessment of coagulation is often required in a clinical setting (Ho and Pavey, 2017). Viscoelastic tests like thromboelastography and thromboelastometry determine coagulation properties of whole blood samples, thereby measuring activities of both platelets and plasma factors (Whiting et al., 2015). Rotational thromboelastometry (ROTEM) provides insights on formation kinetics, stability, and degradation of blood clots at the bed-side. Various tests using different activators/inhibitors provide an overview over hemostasis and its response to therapy with coagulation factors and blood products. Using ROTEM, a recent murine blood banking study revealed that stored mouse erythrocytes have impaired coagulation properties compared to fresh mouse erythrocytes (Pulliam et al., 2020), contradicting the notion of them being procoagulant due to storage lesions.

The aim of this study was to elucidate the impact of storage of human PRBCs on clot formation as assessed by ROTEM assays in reconstituted blood samples. In the first part we explored the effect of storage duration by sampling of PRBCs from the same units after 2, 4 and 6 weeks, and assessing coagulation properties upon reconstitution with fresh, autologous platelet-rich plasma. In the second part, coagulation properties of AB0- and rhesus-matched PRBCs stored for prolonged periods were compared to those of freshly drawn, autologous erythrocytes. Finally, we studied the effects that MVs accumulating during prolonged storage exert on coagulation properties.

## Materials and methods

### Study participants

The observational volunteer study was approved by the Ethics Committee of the Medical University of Vienna (1043/2015), and was registered at clinicaltrials.gov (NCT02639780). Twelve healthy volunteers (Supplementary Table S1) were enrolled after providing written informed consent. Each volunteer donated 450 ml blood for a PRBC unit. In addition, blood (9 ml) was drawn at 2, 4, and 6 weeks after the initial blood donation to prepare control samples for viscoelastic tests and platelet-rich plasma (PRP) from the same individual.

### Processing and storage of experimental PRBC units

Whole blood was drawn from a cubital vein of the study participants, and processed as described previously by the Austrian Red Cross Blood Donation Services (Prosenz et al., 2019). Briefly, blood was leukodepleted and separated into blood components. All PRBC units were stored in saline-adenine-glucose-mannitol solution according to standard blood banking conditions with uninterrupted cooling at 4°C for up to 42 days. Aliquots of PRBCs were aseptically drawn from storage bags at indicated time points. These PRBC units were used for autologous reconstitution of blood samples for ROTEM experiments in the first part of the study. The PRBC units used in the second part of this study were purchased from the Austrian Red Cross Blood Donation Services and stored as described above for up to 6 weeks.

### Determination of PS exposure in PRBCs

Aliquots were aseptically sampled from PRBC units and centrifuged (5 min at 1000 *g*)*.* Pelleted erythrocytes were washed 3 times in phosphate buffered saline (PBS) by centrifugation (5 min at 1000 *g*), and the supernatants discarded. The erythrocytes were diluted to 2 × 10^7^ cells/mL in PBS containing 7.5 mM glucose and 1 mM EDTA (pH = 7.4). Twenty µL/mL of FITC-coupled lactadherin (Lact-FITC, 83 μg/ml; Haematologic Technologies, Inc.) were added, and samples immediately subjected to flow cytometry (488 nm laser, 30,000 events; FACSCalibur, Becton Dickinson).

### Hemolysis in PRBC units

Hemolysis in the supernatant of PRBC units was assessed by Drabkin’s reagent, containing 1 g/L sodiumbicarbonate, 50 mg/L potassiumcyanide, and 200 mg/L potassiumferricyanide (Sigma Aldrich). 100 µM of supernatant were mixed with 600 µM of reagent, incubated for 30 min at room temperature, the optical density at 540 nm determined in a spectrophotometer, and the amount of hemoglobin calculated against a standard curve.

### Rotational thromboelastometry

(Reconstituted) blood samples were stimulated with calcium and ellagic acid (INTEM) or calcium and tissue factor (EXTEM), according to the manufacturer’s instructions and immediately subjected to viscoelastic coagulation testing by ROTEM delta (Tem International GmbH). Clotting time (CT), clot formation time (CFT), clot firmness 10 min after CT (A10), maximum clot firmness (MCF), and α angle were assessed for each sample.

### Preparation of platelet-rich plasma and control erythrocytes

Freshly drawn blood samples were centrifuged for 1 min at 1000 *g*, supernatants (platelet-rich plasma (PRP)) collected and later used for the reconstitution experiments. Erythrocyte pellets were resuspended in PBS and washed 3 times in PBS (1 min at 3000 *g*). The cells from the erythrocyte pellets were used as fresh autologous control cells for the ROTEM experiments in the second part of the study.

### Preparation of PRBC aliquots

Aliquots were aseptically removed from PRBCs 2, 4, and 6 weeks of cold storage. RBCs were pelleted by centrifugation (1 min at 3000 *g*) and washed 3 times in PBS before further use. In the second part of the study, the supernatant after the first centrifugation step was processed further for MV preparation (see below).

### Preparation of MVs from PRBC units

Microvesicles were prepared from stored PRBC units as previously described (Salzer et al., 2008). Briefly, the supernatant of PRBC units was centrifuged at 16,000 *g* for 30 min at 4°C, and the pellet suspended in PBS. After a short centrifugation step (3000 *g* for 1 min) to remove contaminating cellular debris, the supernatant was separated and centrifuged again at 16,000 x *g* for 30 min. The vesicle pellet was suspended in PBS to result in a 40-fold vesicle concentration (40 x MV) as compared to that of the supernatant in PRBC units.

### Reconstitution of blood samples

Two hundred µL of washed and pelleted erythrocytes from PRBCs were combined with an equal volume of freshly prepared autologous PRP to give a reconstituted blood sample with a hematocrit of 50%. Similarly, in the second part of the study, freshly prepared, autologous, packed erythrocytes or heterologous, AB0- and rhesus-matched PRBCs were combined with an equal volume of “modified” PRP preparations resulting in a reconstituted blood sample with a hematocrit of 50%. In the modified PRPs, either 5% and 25% of the volume were replaced by PBS (PRP1 and PRP2, controls) or by a 40 x MV vesicle suspension that result in MV concentrations in the reconstituted blood sample equal or in 5 times excess to that measured in the supernatant of stored PRBC units (1 x MV and 5 x MV, respectively).

### Statistical analysis

Statistical analyses were performed using SPSS 25 (IBM Statistics) and Prism 6 (GraphPad Software, Inc.). Changes of PS exposure and hemolysis over time were analyzed using one-way ANOVA with multiple comparison testing. Pairwise comparisons were performed by paired Student’s t-test.

## Results

**FIGURE 1:**
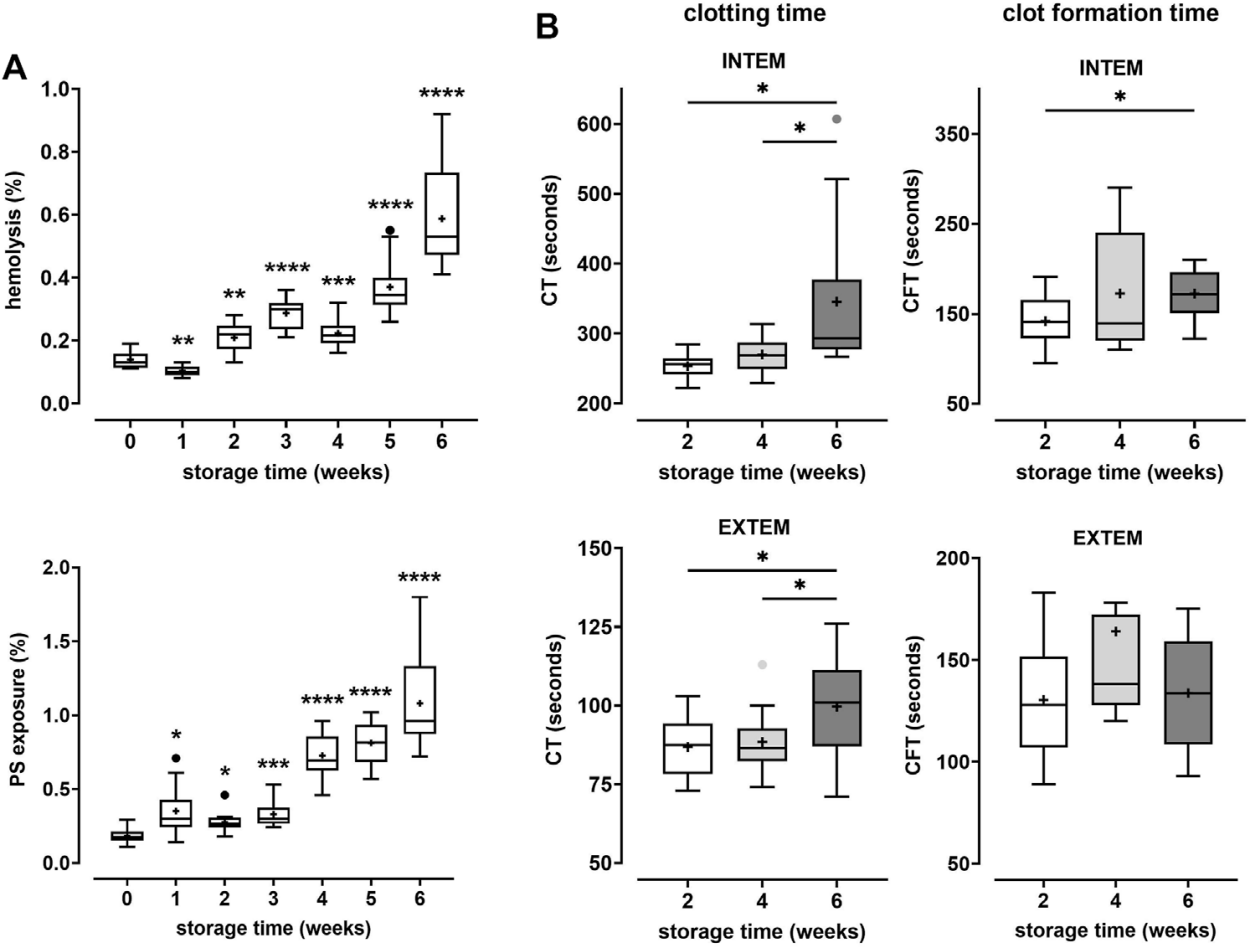
Coagulation properties of PRBCs are progressively impaired during storage. PRBCs from blood donated by 12 healthy volunteers were stored for up to 6 weeks. **(A)** Aliquots were removed weekly and tested for the percentage of erythrocytes that had undergone hemolysis (top panel) or that exposed phosphatidylserine (PS) (bottom panel), respectively. Analyses were performed using repeated-measures-ANOVA with Dunnett’s multiple comparison. Significant changes from base values (week 0) are indicated for *p* < 0.05 *, *p* < 0.01 **, *p* < 0.001 *** and *p* < 0.0001 ****. **(B)** In weeks 2, 4 and 6, aliquots drawn from PRBC units were recombined with autologous platelet-rich plasma (PRP) prepared from fresh blood donations and tested by thromboelastometry applying both INTEM and EXTEM assays. Clotting times (CT) and clot formation times (CFT) are shown in Box Whisker blots with mean values indicated by crosses. Statistically significant differences were assessed by paired t-tests and indicated by stars (*p ≤ 0.05). Numerical values of the data in Figure 1B are given in Table 1.

In this study we addressed the question whether prolonged storage affects coagulation properties of erythrocytes. For this purpose, erythrocytes collected from PRBC units were combined with freshly prepared, autologous or heterologous matched PRP. The coagulation properties of these reconstituted blood samples were assayed by ROTEM. In the first part of the study, PRBC units were prepared from blood donated by healthy participants and stored for up to 42 days at 4°C. Hemolysis and the number of cells with phosphatidylserine (PS) exposed at their surface were assessed on a weekly basis. In line with previous studies (Lu et al., 2011; Dinkla et al., 2014; Mittag et al., 2015), both markers significantly increased during storage. At 6 weeks, 0.59 ± 0.16% of cells in PRBCs had hemolyzed and 1.08 ± 0.32% of cells exposed PS at the outer membrane leaflet (Figure 1A). After 2, 4 and 6 weeks, fresh blood samples were obtained from the participants to prepare PRP. Aliquots from the PRBC units were drawn, cell pellets prepared, washed and combined with autologous PRP to give reconstituted blood samples of 50% hematocrit. Coagulation properties of these samples were assessed in INTEM and EXTEM ROTEM assays. Under these conditions, CTs increased from 253 ± 19 to 346 ± 109 s in INTEM assays and from 87 ± 10 to 100 ± 17 s in EXTEM assays between weeks 2 and 6 of storage, respectively (Figure 1B). The increase in CT values was significant both between week 2 and 6 and between week 4 and 6. Further, CFTs significantly increased between week 2 and 6 in INTEM assays (Figure 1B; Table 1). These data implicate that prolonged storage either diminishes prothrombotic properties or induces antithrombotic properties in PRBCs. This was unexpected since the fraction of PS positive cells - a potential procoagulant factor - was significantly higher in PRBCs at week 6 than at week 2 (Figure 1A). To evaluate the effect of blood fractionation and recombination on coagulation parameters, control blood samples reconstituted from freshly prepared RBCs and PRP to yield 50% hematocrit were compared with unprocessed blood samples. Reconstituted samples revealed similar CTs but considerably increased CFT values (Supplementary Table S2) both in INTEM and EXTEM assays. The latter might be explained by some loss in platelet counts during PRP preparation.

**TABLE 1:** Storage time-dependent change in coagulation characteristics in blood samples reconstituted with PRBCs.

A				
Parameter	Assay	Week 2	Week 4	Week 6
		avg ± stdev (s)	avg ± stdev (s)	avg ± stdev (s)
Clotting time (CT)	INTEM	253 ± 19	270 ± 24	346 ± 109
	EXTEM	87 ± 10	88 ± 10	100 ± 17
Clot formation time (CFT)	INTEM	142 ± 28	173 ± 67	173 ± 27
	EXTEM	130 ± 28	164 ± 61	134 ± 27

**B**				
**Parameter**	**Assay**	**Difference**		**Significance**
		**avg ± stdev (s)**	**%**	***p* value**
**Comparison week 2 vs week 4**				
Clotting time (CT)	INTEM	−17 ± 29	−7%	0.078
	EXTEM	−2 ± 15	−2%	0.728
Clot formation time (CFT)	INTEM	−31 ± 61	−22%	0.108
	EXTEM	−34 ± 59	−26%	0.075
**Comparison week 2 vs week 6**				
Clotting time (CT)	INTEM	−92 ± 115	−36%	**0.018**
	EXTEM	−13 ± 17	−15%	**0.022**
Clot formation time (CFT)	INTEM	−31 ± 35	−22%	**0.011**
	EXTEM	−3 ± 27	−2%	0.680
**Comparison week 4 vs week 6**				
Clotting time (CT)	INTEM	−76 ± 115	−28%	**0.044**
	EXTEM	−11 ± 15	−13%	**0.026**
Clot formation time (CFT)	INTEM	0 ± 65	0%	0.997
	EXTEM	30 ± 56	18%	0.085

PRBC units were prepared from blood donated by 12 healthy volunteers and stored for 6 weeks. At weeks 2, 4 and 6, aliquots from the PRBC units were combined with autologous platelet rich plasma (PRP) prepared from fresh blood donations and tested by thromboelastometry applying both INTEM and EXTEM assays.

A) The mean values (AVG) and standard deviations (STDEV) of the clotting times (CT) and clot formation times (CFT) are given in seconds (s).

B) Pairwise comparisons between weeks are shown as indicated. The mean differences are given in absolute numbers (AVG ± STDEV in s) and in relative percent (compared to the value of the earlier time point). Negative differences indicate a loss, whereas positive differences indicate an increase in coagulability. The significance of the differences [*p*-values (indicated in bold when p<0.05)] was assessed by paired t-tests. The sample size is n = 12.

In the second part of the study, we modified the reconstitution scheme by comparing fresh, autologous erythrocytes and heterologous erythrocytes harvested from ABO- and rhesus-matched PRBCs after 5 or 6 weeks of storage. Moreover, to assess the effect of MVs that accumulate in PRBC units on coagulation properties, MVs were prepared from the supernatant of PRBCS, and were added to reconstituted samples. In these samples, fractions of PRP were replaced by corresponding amounts of MV suspensions to achieve final MV concentrations equivalent (1 x MV) or in 5 times excess (5 x MV) to that measured in the supernatant of stored PRBC units. Control samples had the respective fractions of PRP replaced by PBS (PRP1 and PRP2).

**FIGURE 2:**
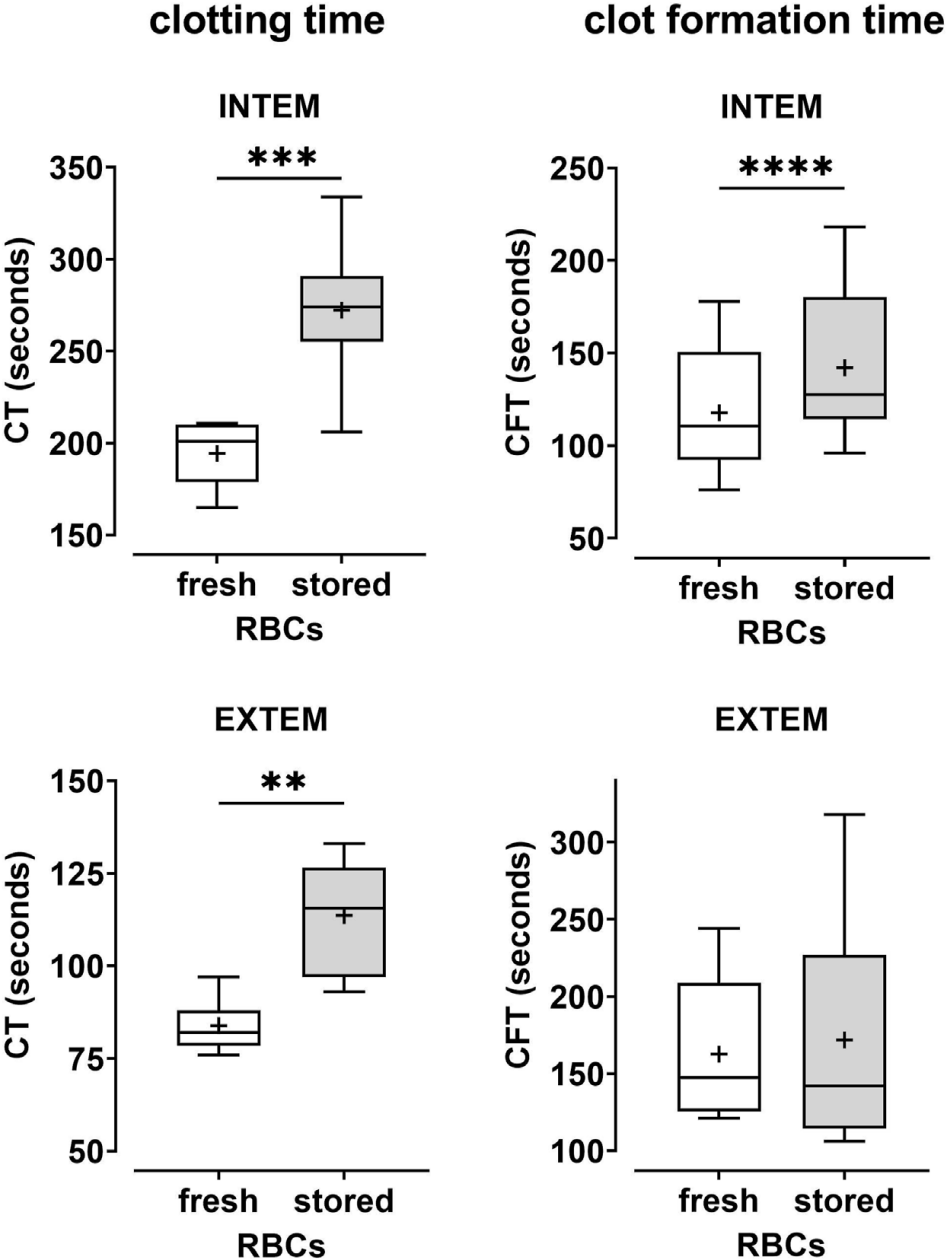
Coagulability is reduced in samples reconstituted with erythrocytes from stored PRBCs as compared to fresh erythrocytes. Freshly prepared PRP (PRP1 as described in the text) from healthy donors was combined with fresh autologous erythrocytes or with erythrocytes from AB0- and rhesus-matched PRBC units that had been stored for 5–6 weeks. Reconstituted blood samples were tested by thromboelastometry applying both INTEM and EXTEM assays. Clotting times (CT) and clot formation times (CFT) are shown in Box Whisker blots with the mean values indicated by crosses. Statistically significant differences were assessed by paired t-tests and indicated by stars (**p ≤ 0.01; ***p ≤ 0.001). Numerical values are given in Table 2 (note that this data set corresponds to blood samples reconstituted with PRP1).

As compared to fresh, autologous RBCs, stored ABO-matched heterologous RBCs collected from PRBCs and reconstituted with PRP1 (Figure 2; Table 2) or PRP2 (Table 2) revealed increased CT and CFT values both in INTEM and EXTEM assays. Apart from CFT in EXTEM assays, the effects were considerable (20%–41% increase in CT and CFT values, respectively) and significant. Similarly, a paired comparison of erythrocytes from stored PRBCs *versus* erythrocytes from fresh blood reconstituted with MV-supplemented PRP (see below) showed significantly increased CTs and CFTs, this time also in CFT EXTEM (Table 2). Together, these data indicate that erythrocytes from stored PRBCs loose some inherent coagulation-promoting property upon storage for 5–6 weeks as compared to fresh autologous erythrocytes.

**TABLE 2:** Coagulation parameters in blood samples reconstituted with erythrocytes from stored PRBCs compared to fresh erythrocytes.

Parameter	Assay	Erythrocytes re-constituted with	Fresh erythrocytes	Stored erythrocytes		Difference		Significance
			avg ± stdev (s)	avg ± stdev (s)	n	avg ± stdev (s)	avg (%)	*p* value
clotting time (CT)	INTEM	PRP1	194 ± 17	272 ± 36	8	−78 ± 37	−40%	**0.001**
		PRP2	208 ± 27	293 ± 77	7	−85 ± 81	−41%	**0.031**
		1xMV	189 ± 15	270 ± 29	8	−82 ± 33	−44%	**0.001**
		5xMV	197 ± 18	293 ± 77	7	−96 ± 92	−49%	**0.032**
	EXTEM	PRP1	84 ± 7	114 ± 16	8	−30 ± 17	−36%	**0.002**
		PRP2	74 ± 6	94 ± 9	7	−19 ± 6	−26%	**0.001**
		1xMV	85 ± 11	104 ± 16	8	−19 ± 16	−22%	**0.016**
		5xMV	77 ± 5	99 ± 10	7	−22 ± 12	−29%	**0.003**
clot formation time (CFT)	INTEM	PRP1	118 ± 35	142 ± 42	8	−24 ± 8	−20%	**0.001**
		PRP2	127 ± 23	152 ± 28	7	−25 ± 19	−20%	**0.014**
		1xMV	132 ± 39	160 ± 49	8	−28 ± 17	−21%	**0.002**
		5xMV	151 ± 33	164 ± 28	7	−14 ± 23	−9%	0.163
	EXTEM	PRP1	163 ± 47	172 ± 75	8	−9 ± 32	−6%	0.437
		PRP2	151 ± 33	158 ± 37	7	−8 ± 8	−5%	0.051
		1xMV	153 ± 54	179 ± 68	8	−26 ± 18	−17%	**0.005**
		5xMV	151 ± 37	170 ± 43	7	−19 ± 14	−13%	**0.010**

Fresh erythrocytes or erythrocytes from PRBCs stored for 5–6 weeks were combined with 4 different sets of platelet rich plasma: PRP that had a fraction of 5% or 25% replaced by PBS (PRP1 and PRP2, respectively) and PRP that had a fraction of 5% or 25% replaced by vesicle concentrate (1 x MV and 5 x MV, respectively). These sets of reconstituted blood samples were tested by thromboelastometry applying both INTEM and EXTEM assays. The mean values (AVG) and standard deviations (STDEV) of the clotting times (CT) and clot formation times (CFT) are given in seconds (s). The mean differences are given in absolute numbers (AVG ± STDEV in s) and in relative percent (compared to the value of fresh erythrocytes). Negative differences indicate a loss of coagulability of stored as compared to fresh erythrocytes. The significance of the difference [*p*-values (indicated in bold when *p* < 0.05)] between fresh and stored erythrocytes was assessed by paired T tests. The sample size (n) is given for each set of experiments.

**FIGURE 3:**
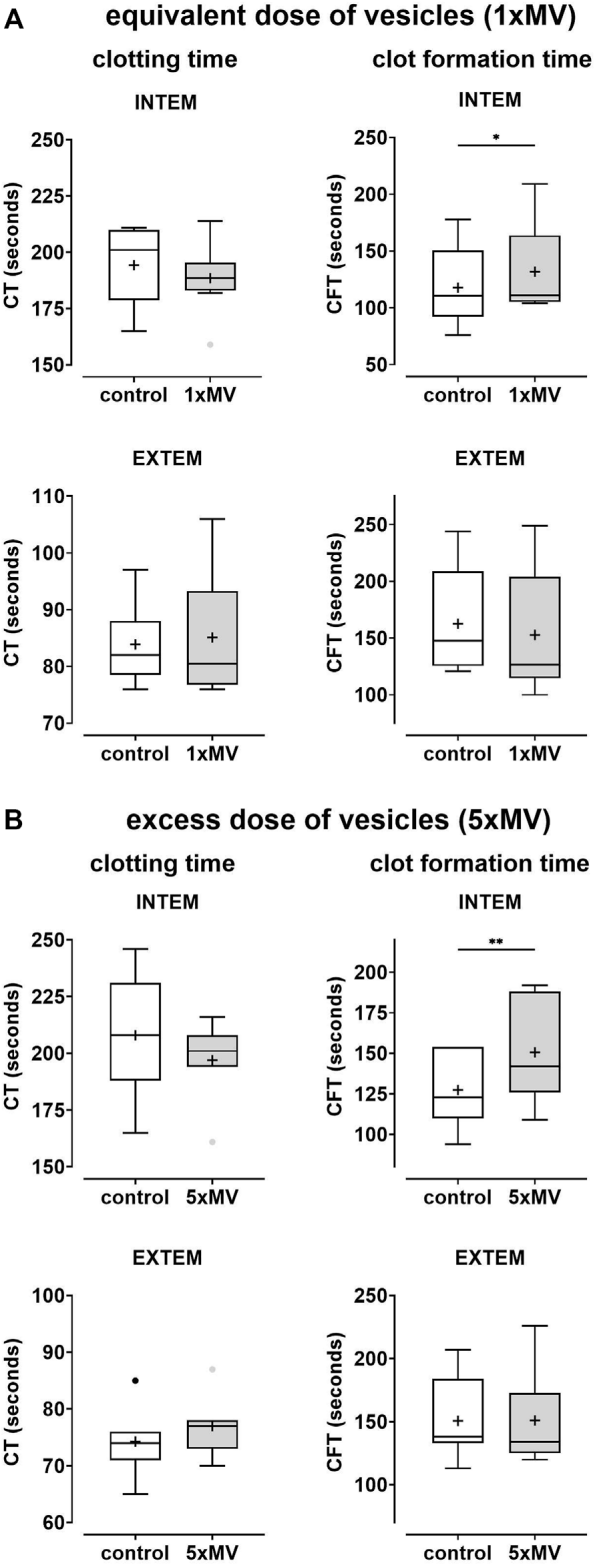
Microvesicles from the supernatant of PRBC units elicit only minor effects on coagulability of reconstituted blood samples. Microvesicles (MVs) isolated from the supernatant of PRBC units. Platelet-rich plasma (PRP) was prepared from freshly drawn blood samples and fractions (5% or 25%) of the PRP were replaced by MV suspensions to give final MV concentrations equivalent (1 x MV) and in 5 times excess (5 x MV) to that in the PRBC unit, **(A**, **B)** respectively. Control samples had the respective fractions of PRP replaced by PBS (PRP1 and PRP2). Blood samples reconstituted from these PRP preparations and fresh autologous erythrocytes were tested by thromboelastometry applying both INTEM and EXTEM assays. Clotting times (CT) and clot formation times (CFT) are shown in Box Whisker blots with the mean values indicated by crosses. Statistically significant differences were assessed by paired t-tests and indicated by stars (*p ≤ 0.05; **p ≤ 0.01). Numerical values are given in Table 3A,B (note that these data sets correspond to blood samples reconstituted with fresh RBCs).

Lastly, to assess the contribution of PRBC-derived MVs on coagulation, blood samples reconstituted with PRP1 and PRP2 were compared with those reconstituted with 1 x MV and 5 x MV, respectively. In samples with fresh erythrocytes, the presence of MVs did not significantly alter CT values in INTEM or EXTEM assays (Figure 3; Table 3A). A significantly decreased CT value (reduced by 13% in vesicle-containing samples) was only seen in erythrocytes from stored PRBCs combined with 5 x MV (Table 3B). In contrast, INTEM CFT values were significantly increased in 1 x MV and 5 x MV samples, both when combined with fresh erythrocytes and stored erythrocytes from PRBC units. Samples with fresh erythrocytes revealed CFT times prolonged by 12% and 18% in the presence of equivalent doses or excess doses of MVs, respectively (Table 3A,B). Stored erythrocytes from PRBCs combined with MVs had the respective CFT times prolonged by 12% and 8% (Table 3A,B). These findings reveal non-uniform effects of MVs on the coagulation process and, unexpectedly, a bias towards an anti-hemostatic impact of MVs in the reconstituted samples.

**TABLE 3:** Effects of vesicles from PRBC units on coagulability of reconstituted blood samples.

A	Parameter	Assay	PRP1/1xMV re-constituted with	PRP1	1xMV		Difference		Significance
				avg ± stdev (s)	avg ± stdev (s)	n	avg ± stdev (s)	avg (%)	*p* value
	clotting time (CT)	INTEM	fresh erythrocytes	194 ± 17	188 ± 15	8	6 ± 17	3%	0.362
			stored erythrocytes	272 ± 36	270 ± 29	8	2 ± 23	1%	0.794
		EXTEM	fresh erythrocytes	84 ± 7	85 ± 11	8	−1 ± 10	−1%	0.737
			stored erythrocytes	114 ± 16	104 ± 16	8	10 ± 16	9%	0.126
	clot formation time (CFT)	INTEM	fresh erythrocytes	118 ± 35	132 ± 39	8	−14 ± 15	−12%	**0.030**
			stored erythrocytes	142 ± 42	160 ± 49	8	−17 ± 13	−12%	**0.006**
		EXTEM	fresh erythrocytes	163 ± 47	153 ± 54	8	10 ± 22	6%	0.241
			stored erythrocytes	172 ± 75	179 ± 68	8	−7 ± 43	−4%	0.650

B	Parameter	Assay	PRP2/5xMV re-constituted with	PRP2	5xMV		Difference		Significance
				avg ± stdev (s)	avg ± stdev (s)	n	avg ± stdev (s)	avg (%)	*p* value
	clotting time (CT)	INTEM	fresh erythrocytes	208 ± 27	197 ± 18	7	11 ± 24	5%	0.277
			stored erythrocytes	293 ± 77	256 ± 65	7	38 ± 26	13%	**0.009**
		EXTEM	fresh erythrocytes	74 ± 6	77 ± 5	7	−3 ± 6	−4%	0.306
			stored erythrocytes	94 ± 9	99 ± 10	7	−5 ± 9	−5%	0.192
	clot formation time (CFT)	INTEM	fresh erythrocytes	127 ± 23	151 ± 33	7	−23 ± 13	−18%	**0.003**
			stored erythrocytes	152 ± 28	164 ± 28	7	−12 ± 3	−8%	**0.001**
		EXTEM	fresh erythrocytes	151 ± 33	151 ± 38	7	0 ± 12	0%	0.930
			stored erythrocytes	158 ± 37	170 ± 43	7	−12 ± 9	−8%	**0.013**

Microvesicles (MVs) isolated from the supernatant of PRBC units were concentrated 40 times in PBS. Platelet-rich plasma (PRP) was prepared from freshly drawn blood samples and fractions (5% or 25%) of the PRP were replaced by MV suspensions to give final MV concentrations equivalent (1 x MV) and in 5 times excess (5 x MV) to that in the PRBC unit, A and B respectively. Control samples had the respective fractions of PRP replaced by PBS (PRP1 and PRP2). Blood samples were reconstituted by combining these PRP preparations with either fresh autologous erythrocytes or stored, AB0- and rhesus-matched erythrocytes from stored PRBC units (as indicated) and tested by thromboelastometry applying both INTEM and EXTEM assays. The mean values (AVG) and standard deviations (STDEV) of the clotting times (CT) and clot formation times (CFT) are given in seconds (s). The mean differences are given in absolute numbers (AVG ± STDEV in s) and in relative percent. Negative differences indicate a loss whereas positive differences indicate an increase in coagulability of MV-supplemented as compared to control-supplemented blood samples. The significance of the differences [*p*-values (indicated in bold when *p* < 0.05)] was assessed by paired t-tests. The sample size (n) is given for each set of experiments.

## Discussion

Using rotational thromboelastometry (ROTEM) as a tool to more closely mimic in vivo-like conditions, the prime finding of our study is that storage-associated changes in erythrocytes from stored PRBCs alter inherent coagulation properties. This finding was validated 1) in the time course experiment comparing PRBCs stored for longer *versus* shorter periods upon reconstitution with autologous PRP (Figure 1B; Table 1) and 2) in the comparison of fresh autologous RBCs with heterologous, AB0- and rhesus-matched erythrocytes from PRBCs stored for prolonged periods (Figure 2; Table 2). Clotting times were significantly prolonged upon storage for 6 weeks (as compared to 2 weeks) despite the higher proportion of PS-positive cells in these PRBCs (Figure 1A). Surface-exposed PS is generally considered as a procoagulant factor promoting assembly of components of the coagulation cascade, thereby accelerating thrombin generation. Thus, our findings implicate that the PS-dependent procoagulant contribution is more than outweighed by an anticoagulant property of PRBCs attained upon prolonged storage. This anticoagulant property of erythrocytes from stored PRBCs became even more evident when these were compared to fresh erythrocytes by ROTEM, revealing significant effects on CTs and CFTs (Figure 2 and Table 2). The latter suggests that some process involving the interaction with fibrin fibers and/or platelets likely deteriorates during prolonged storage of PRBCs. Our data are in line with recent findings from a murine blood banking study describing a loss in coagulability of aged PRBCs (Pulliam et al., 2020), thereby implicitly corroborating the emerging view that RBCs play an active role in physiological hemostasis (Weisel and Litvinov, 2019). At present, we can only speculate about molecular mechanisms mediating the functional involvement of erythrocytes in coagulation, but the procoagulant response of erythrocytes upon exposure to platelet-derived lysophosphatidic acid (LPA) can be considered as a potential factor (discussed further below).

The second result of our study was the observation that MVs shed by erythrocytes during prolonged storage have a rather small impact on coagulation when present in the reconstituted samples. The only statistically significant prothrombotic effect was observed when MVs were added to PRBC samples in 5 times excess resulting in a shortening of the clotting time by 13% in INTEM assays (Table 3B). While this particular finding is in line with the prevailing view on MVs (PS-rich vesicular surfaces accelerating thrombin generation) it vanishes when MVs were added to samples in amounts that reflect the vesicle/erythrocyte ratios in the PRBC unit. In contrast, the presence of vesicles (both in equivalent and excess amounts) significantly prolonged CFT in INTEM assays (Figure 3; Table 3). This finding suggests that vesicles disturb the process of clot formation probably by interfering with the interactions between erythrocytes and platelets and/or the crosslinking of fibrin filaments. A recent study by Fischer et al. reported a reduction in clotting times in INTEM assays by 18% when vesicles from the supernatant of PRBC units were added to blood samples (Fischer et al., 2017). They also observed an - albeit statistically not significant–increase in CFT values. Although roughly in line with our findings, it is difficult to directly compare results of both studies due to differences in vesicle preparation and blood sample composition.

There is currently no consensus as to the effect of transfusion on hemostasis. Several studies report an association between PRBC transfusion and increased venous thromboembolic risk, suggesting a procoagulant effect (Goel et al., 2018; Rothstein et al., 2019). However, in non-variceal gastrointestinal hemorrhage, blood transfusion within 24 h of presentation was associated with increased mortality (Taha et al., 2011) and specifically with an increased risk of re-bleeding (Restellini et al., 2013), suggesting a possible anti-hemostatic effect. However, this effect could also be attributed to increased portal vein pressures following transfusion. Conceivably, pro- and anti-hemostatic effects of PRBC transfusions involve different mechanisms with potentially detrimental effects depending on the pathological background of the recipient. One can envisage hypothetical scenarios where blood transfusions may be associated with either increased bleeding or an enhanced thromboembolic risk. Physiological clotting requires the concerted interplay of plasmatic with cellular factors resulting in a rapid, avalanche-like, yet controlled process of local clot formation. Assuming a central role of erythrocytes in this process, a high responsiveness of erythrocytes to local triggers like platelet-secreted LPA may be crucial. The fast presentation of PS at the surface of erythrocytes upon exposure to LPA and the efficient release of MVs could be essential to initiate and confine clot formation at/to the site of vascular injury. PRBC storage lesions could affect the responsiveness of erythrocytes by reducing the efficacy of their intracellular signaling for rapid PS exposure and the membrane flexibility for vesicle shedding. This, however, is no contradiction to the fact that a fraction of erythrocytes in aged PRBCs already expose PS at their surface. Distributing such erythrocytes and MVs systemically throughout the circulatory system may sequester some clotting factors and - by lowering their availability - may hamper rather than promote local physiological clot formation. This could possibly explain transfusion-associated re-bleeding in non-variceal gastrointestinal hemorrhage. On the contrary, MVs from aged PRBCs were shown to induce pulmonary microthrombus formation in a mouse model (Kim et al., 2020). Part of the prothrombotic activity was mediated by endothelial cells activated by vesicle exposure. Similarly, MVs from PRBC units can elicit effects on neutrophils, platelets and monocytes (Jank and Salzer, 2011; Kent et al., 2014; Fischer et al., 2017). Thus, one may assume that, upon transfusion, MVs become absorbed by endothelial and blood cells, and their thrombogenic effect is mainly exerted indirectly *via* activation of these cells rather than by direct participation in coagulation. Moreover, procoagulant MVs are not only produced during storage, but also after transfusion within the recipient. This is corroborated by results from a recent study using a model of recipient-mimicking conditions. After *in vitro* reconstitution of stored PRBCs with recipient blood, PS-positive MVs progressively increased over time. Clearly, these transfusion-induced MVs were not experimentally accessible in our study (Tzounakas et al., 2022).

Two limitations of this ROTEM-based study should shortly be addressed: 1) even though ROTEM analyses entail a broad assessment of coagulation properties, hemostasis *in vivo* is also influenced by vasculature, blood cells and hemodynamic factors. These interactions are experimentally inaccessible by ROTEM, thereby posing a general limit to these analyses in simulating coagulation processes *in vivo*. 2) The experimental approach of this study required the fractionation and preparation of blood components (fresh PRP, fresh erythrocytes, erythrocytes from stored PRBCs and MVs) and the reconstitution of these components into “artificial” blood samples. Comparing ROTEM values of fresh unprocessed blood with that of samples reconstituted from fresh erythrocytes and PRP revealed similar clotting times, yet increased clot formation times (Supplementary Table S2) both in INTEM and EXTEM assays, indicating a modulation of coagulation properties in reconstituted samples. However, a pairwise comparison with respective direct controls allowed to dissect the relative influences of RBC storage and MVs on the coagulation process. ROTEM analyses of experimentally reconstituted blood samples could and should further be exploited to address the effects of other blood cells, platelet counts and plasma factors on coagulation and may thereby be established as a valuable complementary approach in hemostasis research.

Taken together, our results and these considerations suggest that transfusion of PRBC units stored for prolonged periods might affect two rather independent phenomena: a thrombogenic effect due to activation of cells in the circulatory system by co-transfused MVs and an anti-hemostatic effect due to changes in erythrocytes from stored PRBCs that impair their physiological contribution to coagulation. This can be either viewed as loss of a physiologic erythrocyte function in coagulation or as a storage-induced gain in a coagulation-retarding property. Future studies will have to delineate molecular mechanisms that enroll erythrocytes in physiologic hemostasis and the alteration of this function in erythrocytes from stored PRBC units. This functional alteration, however, should be added to the list of changes in PRBCs that are commonly addressed as “storage lesions”.

## Data Availability

The original contributions presented in the study are included in the article/Supplementary Material, further inquiries can be directed to the corresponding author.

## Supplementary material

SUPPLEMENTARY TABLE S1 Characteristics of blood donors.

SUPPLEMENTARY TABLE S2 Comparison of coagulation properties between whole blood and reconstituted blood samples. Freshly drawn blood samples were either left unprocessed (whole blood sample) or separated in platelet-rich plasma (PRP) and erythrocytes, and further recombined in a 1:1 volume ratio (reconstituted blood sample). These blood samples were assessed by thromboelastometry applying both INTEM and EXTEM assays. Clotting time (CT) and clot formation time (CFT) are given in seconds, A10 and MCF in millimeters and α angle in degree°. The results are given in mean values ± standard deviations. Significant differences between the samples are indicated in bold with *p*-values ≤ 0.05.
