# Supplementary material for: Storage of packed red blood cells impairs an inherent coagulation property of erythrocytes
Source: Front Physiol. 2022 Nov 25;13:1021553. doi: 10.3389/fphys.2022.1021553 (PMC9732456; doi:10.3389/fphys.2022.1021553)
Supplement: Supplementary file 1 [file Table1.docx]

| **characteristic** | **value** |
| --- | --- |
| age (years), mean [IQR] | 44 [36-58] |
| **gender** |  |
| female, n (%) | 5 (42) |
| male, n (%) | 7 (58) |
| **blood group** |  |
| A positive, n (%) | 4 (33) |
| A negative, n (%) | 2 (17) |
| O positive, n (%) | 6 (50) |
| hemoglobin donor (g/dl) | 15±1 |
| hemoglobin PRBCs (g/dl) | 21±1 |
