# Supplementary material for: Storage of packed red blood cells impairs an inherent coagulation property of erythrocytes
Source: Front Physiol. 2022 Nov 25;13:1021553. doi: 10.3389/fphys.2022.1021553 (PMC9732456; doi:10.3389/fphys.2022.1021553)
Supplement: Supplementary file 2 [file Table2.docx]

|  |  | **fresh** | | **reconstituted** | | **p-value** | |  |
| --- | --- | --- | --- | --- | --- | --- | --- | --- |
| INTEM | CT (s) | | 196 ± 7 | | 194 ± 17 | | 0.842 | |
|  | A10 (mm) | | 57 ± 6 | | 42 ± 7 | | **0.001** | |
|  | CFT (s) | | 90 ± 29 | | 176 ± 77 | | **0.041** | |
|  | MCF (mm) | | 64 ± 6 | | 51 ± 4 | | **0.001** | |
|  | α angle (°) | | 74 ± 3 | | 67 ± 2 | | **0.018** | |
| EXTEM | CT (s) | | 71 ± 9 | | 69 ± 13 | | 0.719 | |
|  | A10 (mm) | | 59 ± 6 | | 45 ± 8 | | **0.013** | |
|  | CFT (s) | | 80 ± 12 | | 157 ± 78 | | 0.069 | |
|  | MCF (mm) | | 66 ± 5 | | 53 ± 8 | | **0.011** | |
|  | α angle (°) | | 74 ± 4 | | 65 ± 6 | | **0.024** | |
